# A rare entity to major outbreak: a case report on mucormycosis

**DOI:** 10.11604/pamj.2021.39.183.30479

**Published:** 2021-07-07

**Authors:** Dhaman Gupta, Tanvi Dosi

**Affiliations:** 1 Department of Public Health Dentistry, Sri Aurobindo College of Dentistry, Indore, Madhya Pradesh, India,; 2 Department of Oral Medicine and Radiology, Sri Aurobindo College of Dentistry, Indore, Madhya Pradesh, India

**Keywords:** Mucormycosis, diabetes mellitus, COVID-19, case report

## Abstract

Mucormycosis is relatively uncommon, fulminant, progressive, life threatening fungal disease which is most often seen in debilitating patients with immunocompromised condition. Mucormycosis cases are seen in patients with the use of systemic steroids in the treatment of severely affected COVID-19 cases and also in the patients with uncontrolled diabetes which causes immunosuppression are being reported with mucormycosis. The main symptoms of this disease include pain on the temporal and the orbital region of the affected side which could be throbbing or lancinating type, mobility of the teeth, jaw pain and often swelling is present which could be extraoral and intraoral both or sometimes only intraorally. The diagnostic approach in such cases is done with the help of clinical diagnosis, histopathology and with advanced imaging like cone beam computed tomography, magnetic resonance imaging and computed tomography. We here used cone beam computed tomography imaging that revealed haziness in the sinuses and breach in cortical bone of the affected area which confirmed the diagnosis of mucormycosis. Early treatment planning like administration of antifungal drugs and surgical debridement will be life saving in such a deadly disease.

## Introduction

Mucormycosis is a fungal disease associated with long term morbidity and mortality [1,2]. It is caused by fungi of the order mucorales [3]. After candidiasis and aspergillosis, mucormycosis is the third most common fungal infection [4,5]. This disease was described by Furbinger in 1976 and first case was published by Arnold Paltauf in 1885 [6]. Around 27 species and eleven genera under mucorales are associated with infections in humans. Mostly mucormycosis is caused by rhizopus arrhizus followed by apophyromyces, lichtheimia, mucor, rhizomucor and cunninghamella species [7]. Port of entry of mucormycosis is by direct contamination of open oral wounds or by inhalation of spores [8]. Mucormycosis commonly affects orbit, nose, paranasal sinuses and central nervous system [9]. COVID-19 pandemic continues to be a major problem around the world. Though various options have been used but systemic steroids found to be effective in the survival of COVID-19 [10]. But the use of systemic steroids can lead to fungal or secondary bacterial infection [11]. High prevalence of diabetes patients are seen in India [12]. Systemic steroid and underlying condition like uncontrolled diabetes after recovering from COVID-19 are well known risk factors for mucormycosis reported in literature. This paper reports two such cases.

## Patient and observation

### Case 1

**Information for the patient:** a 58-year-old male, reported to the outpatient department, having pain in the right maxillary quadrant. Pain was continuous and lancinating in nature and radiated to temporal region resulting in a headache. Patient suffered from COVID-19 a month ago. Patient took medications for 5 days along with steroid therapy. There was history of diabetes since 4 years.

**Clinical results:** on extra-oral examination, there was a diffuse swelling on the left side of the face which was tender on palpation. Pertaining to the pain, patient had undergone extraction of right maxillary third molar, 20 days ago with no pain relief; ruling out the possibility of the tooth being the cause of pain. Intra-oral examination revealed mobility in all the teeth of first quadrant. Other intraoral findings included inflammation of palatal gingiva extending up to the mid-palatine raphe which was tender when palpated and exhibited the cardinal signs of inflammation.

**Figure 1:**
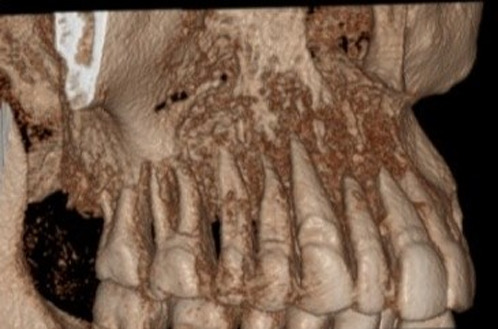
three-dimensional view showing bony destruction in right maxillary quadrant

**Figure 2:**
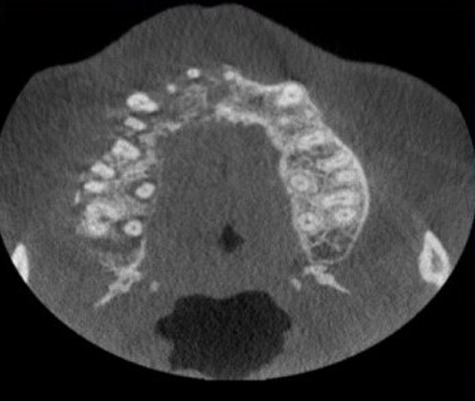
axial cone beam computed tomography (CBCT) view shows loss of buccal cortical bone

**Figure 3:**
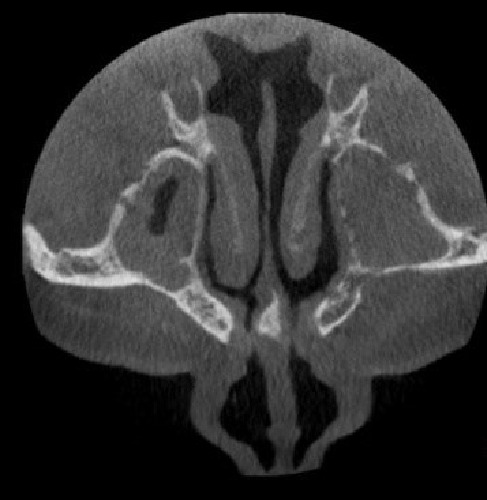
bone destruction and haziness in maxillary sinus shown in axial cone beam computed tomography (CBCT) imaging

**Diagnostic procedure:** cone beam computed tomography (CBCT) revealed; breach in the buccal and palatal cortical bone and illustrated scattered hypodense areas in the right anterior and posterior region. In conjunction with these findings, there was also evidence of discernible haziness and bone destruction along the floor, lateral wall and medial wall of the right and left maxillary sinus (Figure 1, Figure 2, Figure 3).

**Therapeutic intervention and follow-up:** patient was admitted in the other hospital for surgical debridement and antifungal drug amphotericin B was administered for the patient´s recovery.

### Case 2

**Information for the patient:** a 60-year-old male, reported in the department with the complaint of pain in the right upper posterior teeth region with a diffuse swelling in right facial region. Patient had a history of COVID-19 with 20% lung involvement for which he was admitted. The treatment received included, oxygen therapy and steroid administration for approximately five days. He had diabetes since 7 years. Around 15 days later, he started experiencing pain and mobility in maxillary posterior tooth region which was throbbing type and continuous in nature radiating towards right temporal and orbital region.

**Clinical results:** on examination, diffuse extra oral swelling was present on the right side of face. Extension of the swelling superiorly extending upto infraorbital rim and inferiorly till the inferior border of mandible which was soft in consistency and tender on palpation. On intraoral examination, gingival enlargement was observed with blackish discoloration on facial gingiva posteriorly. Mobility was noted in anterior as well as posterior maxillary teeth.

**Figure 4:**
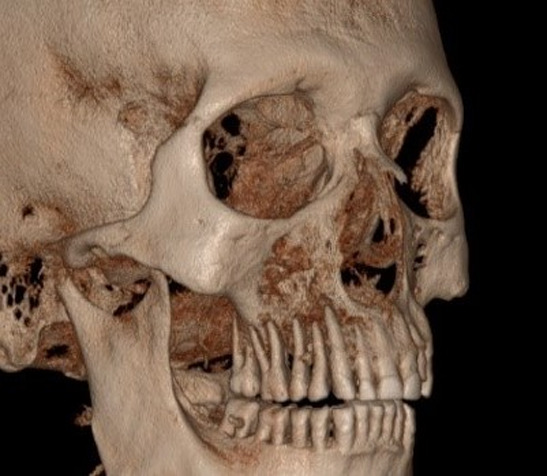
three-dimensional view showing bony destruction in right maxillary quadrant

**Figure 5:**
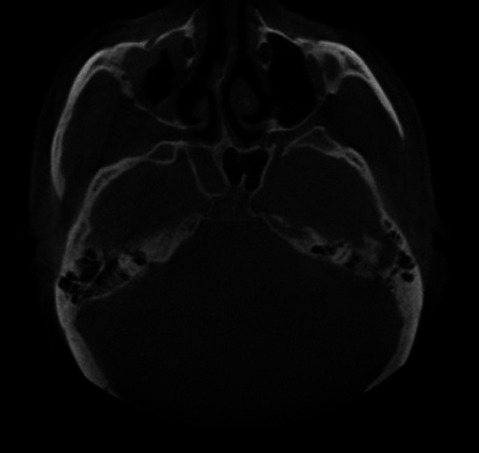
maxillary and sphenoid sinuses showing haziness

**Figure 6:**
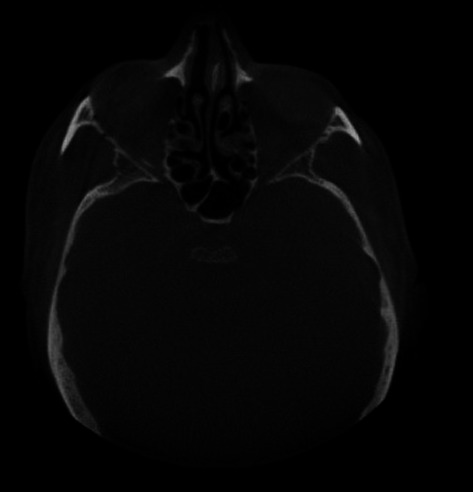
ethmoid sinus showing mild haziness and thickening of sinus lining

**Diagnostic procedure:** cone beam computed tomography (CBCT) was advised as the suitable radiographic imaging modality which revealed scattered hypodensity present in anterior as well as posterior maxillary region along with loss of buccal cortical bone seen in posterior aspect as seen in the axial plane. Paranasal air sinuses involving right maxillary, sphenoid and ethmoid sinuses encased a hazy component giving soft tissue appearance with breaching in medial and lateral wall of maxillary sinus (Figure 4, Figure 5, Figure 6).

**Therapeutic intervention and follow-up:** to attain the better result patient was advised for surgical debridement and antifungal drug amphotericin B which was administered in the other hospital.

## Discussion

World is already facing COVID-19 pandemic, emergence of new outbreak of mucormycosis. Mucormycosis is a rare life threatening black fungal infection associated in patients recovered from COVID-19 [13]. Most of the time, pathogens are found in soil, fruits, manure and dust. It can be obtained from oral cavity, throat, nasal passage and sometimes in healthy patients´ stool also [14]. Port of entry is by direct contamination through skin laceration and by inhalation of spores through mouth or nose [15]. Sino-orbital or pansinusitis type of mucormycosis is found commonly in patients with uncontrolled diabetes and those who are administered with systemic steroids [16,17]. Normally fungi are avirulent; they become virulent when the patients´ immunity becomes low generally happens in immunocompromised patients [1].

Diabetes mellitus also increases fungal proliferation. Patients recovered from COVID-19 having uncontrolled diabetes are known to have significantly low host defence mechanism thus reducing phagocytic and chemotaxis efficiency. Diabetes patients produce keto-reductase enzyme which allow mucormycosis to utilize ketone bodies. Diabetes also alters the activity of transferrin which binds the iron resulting in growth of mucormycosis [8]. Systemic steroids are inexpensive and have shown beneficial effects in COVID-19 patients but also increase the risk of secondary infections [10]. Therefore, both uncontrolled diabetes and steroid use in covid recovering patients are associated factors for pansinusitis or rhino-sinusitis. The symptoms presenting in both the cases of pansinusitis were pain in temporal and orbital region resulting in headache, swelling near infra orbital rim, mobility of teeth, inflammation, enlargement and blackish discoloration of facial gingiva and mild palatal swelling. It initiates from oral and nasal mucosa spreading to all the paranasal sinuses.

To detect the spread and propagation of mucormycosis cone beam computed tomography (CBCT) imaging was used. CBCT revealed areas of hypo density and breach in buccal and cortical bone with evidence of discernible haziness, bone destruction along the floor, lateral and medial wall of right and left maxillary sinus. Sinus thickening and haziness of ethmoid and sphenoid paranasal sinuses were also observed. Surgical debridement and anti fungal drugs like amphotericin B can be administered in such cases to attain the maximum for the patient´s recovery.

## Conclusion

Mucormycosis is a relatively uncommon, fulminant, progressive and life threatening fungal disease mostly occurring in the debilitating patients with immuno compromised condition. It is usually associated with patients having uncontrolled diabetes and the use of systemic steroids in the treatment of severely affected COVID-19 cases. Early diagnosis and treatment of mucormycosis which include antifungal drugs and surgery can improve the survival and outcome.
